# Human-derived Treg and MSC combination therapy may augment immunosuppressive potency in vitro, but did not improve blood brain barrier integrity in an experimental rat traumatic brain injury model

**DOI:** 10.1371/journal.pone.0251601

**Published:** 2021-05-26

**Authors:** Henry W. Caplan, Karthik S. Prabhakara, Naama E. Toledano Furman, Soheil Zorofchian, Cecilia Martin, Hasen Xue, Scott D. Olson, Charles S. Cox

**Affiliations:** Department of Pediatric Surgery, McGovern Medical School, University of Texas Health Science Center at Houston, Houston, Texas, United States of America; Universitatsklinikum Wurzburg, GERMANY

## Abstract

Traumatic brain injury (TBI) causes both physical disruption of the blood brain barrier (BBB) and altered immune responses that can lead to significant secondary brain injury and chronic inflammation within the central nervous system (CNS). Cell therapies, including mesenchymal stromal cells (MSC), have been shown to restore BBB integrity and augment endogenous splenic regulatory T cells (Treg), a subset of CD4+ T cells that function to regulate immune responses and prevent autoimmunity. We have recently shown that infusion of human cord blood-derived Treg decreased neuroinflammation after TBI in vivo and in vitro. However, while both cells have demonstrated anti-inflammatory and regenerative potential, they likely utilize differing, although potentially overlapping, mechanisms. Furthermore, studies investigating these two cell types together, as a combination therapy, are lacking. In this study, we compared the ability of Treg+MSC combination therapy, as well as MSC and Treg monotherapies, to improve BBB permeability in vivo and suppress inflammation in vitro. While Treg+MSC combination did not significantly augment potency in vivo, our in vitro data demonstrates that combination therapy may augment therapeutic potency and immunosuppressive potential compared to Treg or MSC monotherapy.

## Introduction

In the United States alone, there are over 2 million cases of traumatic brain injury (TBI) each year, leading to approximately 50,000 deaths, as well as significant long-term morbidity for survivors [1, 2]. There are two broad phases of TBI: 1) the primary injury itself, which causes immediate tissue damage and neuronal cell death and 2) a hyperexcitatory and inflammatory-mediated secondary brain injury in the subsequent hours to days and beyond [3]. The prevention or mitigation of secondary brain injury has been studied extensively over the preceding decades; however, to date, no therapies have proven effective in late-stage clinical trials [4]. Our lab has found that cell therapies, such as mesenchymal stromal cells (MSC), can mitigate neuroinflammation and improve outcomes in pre-clinical studies [5, 6]. In this study, we specifically examined the ability of cell therapy to restore the blood brain barrier (BBB), as disruption leads to increased edema and decreased effectiveness of osmotherapies to reduce pathologic increases in intracranial pressure. Previous work has demonstrated that MSC effectively improve BBB integrity, likely through up-regulation and organization of tight junctional proteins [7, 8]. Furthermore, we have recently demonstrated that human cord blood-derived regulatory T cell (Treg) therapy decreased chronic neuroinflammation in a rodent model [9]. However, Treg therapy did not improve BBB integrity in our model.

While both MSC and Treg utilize numerous mechanisms to down-regulate inflammatory responses, our lab and others have demonstrated that cross-talk between MSC and Treg appears to play an important role in the efficacy of these therapies [10, 11]. Furthermore, others have shown that MSC and Treg combination therapy can have synergistic effects in vitro and in vivo [12–16]. However, Treg+MSC combination therapy has not been examined as a treatment for TBI. Furthermore, to our knowledge, there are no in vivo studies that have used MSC and Treg combination therapy both derived from human sources. Therefore, in this study, we examined the ability of human MSC+Treg combination therapy, as well as Treg and MSC monotherapies, to improve BBB permeability and attenuate inflammatory responses by rat and human immune cells in vitro. Furthermore, we examined production of Prostaglandin E2 (PGE2) and amphiregulin (AREG), two inflammatory mediators that may contribute to both MSC and Treg functions [17–19]. We hypothesized that combination therapy would confer increased potency and efficacy compared to both Treg and MSC monotherapies. In vivo, Treg+MSC combination did not significantly improve BBB permeability or alter the host immune response compared to monotherapy; however, our in vitro data demonstrated that combination therapy may augment therapeutic potency and immunosuppressive potential.

## Materials and methods

All protocols involving the use of animals were in compliance with the National Institutes of Health Guide for the Care and Use of Laboratory Animals and were approved by the University of Texas Health Science Center Institutional Animal Care and Use Committee (AWC-18-0121). Human peripheral blood was obtained after informed written consent from healthy human adult donors according to a protocol approved by the institutional review board (IRB) (HSC-MS-10-0190). Human umbilical cord blood was obtained via a Material Transfer Agreement (MTA) with the MD Anderson Cord Blood Bank (Houston, TX).

### Animals

Male Sprague Dawley Rats (225–250 g, Envigo Labs) were the source of brain and splenic tissue. The usage of the animals was approved by the Animal Welfare Committee at University of Texas Health Science Center at Houston, Texas, protocol: AWC-18-0121. Animals were handled in accordance with the standards of the American Association for the Accreditation of Laboratory Animal Care (AAALAC). Five-week old rats were housed in pairs under 12 hour light/dark cycles in temperature-controlled conditions. Water and standard rodent laboratory chow were accessible *ad libitum*.

#### 1. Treg isolation, expansion, and characterization

*Human umbilical cord blood mononuclear cell isolation*. Mononuclear cells (MNC) were isolated from fresh human umbilical cord blood (UCB) using 50 mL SepMate-50 PBMC Isolation tubes, as previously described (STEMCELL Technologies, Inc., Vancouver, Canada) [9]. Briefly, 15 mL of Ficoll-Paque density gradient medium (GE Healthcare, Chicago, IL) was added to each tube. Human umbilical cord blood was mixed 1:1 with phosphate buffered saline (PBS) (Lonza, Basel, Switzerland), 30 mL was carefully pipetted on top of the Ficoll-Paque. The tubes were centrifuged for 20 minutes at 1200g with the brake on. The top layer containing the MNC was quickly poured off into another 50 mL centrifuge tube. The cells were washed with PBS and centrifuged at 400g for 8 minutes with the brake on. The cells were then counted and viability was assessed using a NucleoCounter NC-200 and Via1-Cassettes (ChemoMetec, Allerod, Denmark). A repeat wash was performed, and the cells were utilized for subsequent CD4+CD25+ cell isolation.

*Treg isolation and expansion*. Treg were isolated and expanded from fresh UCB MNC as previously described [9]. Briefly, CD4+CD25+ cells were isolated using a human regulatory T cell isolation kit (Miltenyi Biotec, Auburn, CA) and expanded using the MACS GMP ExpAct Treg kit (Miltenyi Biotec) in Treg expansion media, which consisted of HyClone RPMI 1640 (GE Healthcare, Chicago, IL), 5% human AB serum (Thermo Fisher Scientific, Waltham, MA), 1% GlutaMAX (Gibco, Thermo Fisher Scientific, Waltham, MA), 10 μg/mL of gentamicin (Gibco), 100nM Rapamycin (Thermo Fisher Scientific, Waltham, MA), and 500 IU/mL IL-2 (CellGenix, Freiburg, Germany). Cells were split every 2–3 days to maintain a cell density of 5x10^5^ cells/mL and transferred to increasing sized culture vessels based on cell count. The cells were re-stimulated with beads at a ratio of 4:1 bead:cell (ExpAct Treg Beads, Miltenyi Biotec) on day 15 and harvested on day 21. Beads were removed using a MACSiMAG magnet Separator (Miltenyi Biotec). The cells were cryopreserved in 10% HSA and 90% DMSO (Thermo Fisher Scientific, Waltham, MA) at a cell density of 5x10^6^ cells/mL. 1mL of cell suspension was placed in 1.5mL cryovials (Corning, Corning, NY). The cryovials were placed in a Mr. Frosty Freezing Container (Nalgene Nunc, Rochester, NY) and stored at -80°C for 24 hours. They were then transferred to liquid nitrogen vapor phase storage and stored until further use.

*Bone marrow MSC isolation and expansion*. Bone marrow-derived MSC were isolated from a commercially available fresh human bone marrow aspirate (AllCells, Alameda, CA) and expanded following established procedures [8, 20]. Briefly, whole bone marrow aspirates were diluted 1:1 with PBS followed by filtration through a 70μm sieve followed by density centrifugation using SepMate-50 tubes and Ficoll-Paque to isolate the mononuclear cell fraction (“buffy coat”) which was then plated at an initial density of approximately 50,000 cells/cm^2^ on tissue-culture treated T-flasks (Nunc) in in complete culture medium that consisted of alpha‐minimal essential medium (Life Technologies, Grand Island, NY), 17% fetal bovine serum (FBS; lot‐selected for rapid growth of MSC; Atlanta Biologicals, Norcross, GA), 100 units/ml penicillin (Thermo Fisher Scientific, Waltham, MA), 100 mg/ml streptomycin (Life Technologies), and 2 mM l‐glutamine (Thermo Fisher Scientific, Waltham, MA). Non-adherent cells were removed 72 hrs using a gently wash with PBS and replacing the media. MSC were incubated with medium replaced every 2 days until 70% confluent. Medium was then discarded and cultures were washed with phosphate‐buffered saline (PBS) and adherent cells were harvested with 0.25% trypsin/1 mM EDTA (Thermo Fisher Scientific) for 5 minutes at 37°C and additional subcultures were seeded at a density of 1,000 cells/cm^2^ in tissue culture treated T flasks. For cryopreservation, the cells were washed and cryopreserved in a 1:1 dilution of CryoStor CS10 in expansion medium at a density of 2 x 10^6^ cells/cryovial. The cryovials were placed in a Mr. Frosty Freezing Container and stored at -80C overnight. The next day, the cryovials were transferred to liquid nitrogen vapor phase storage and stored until further use.

#### 2. In vitro experiments

*Rat splenocyte isolation and activation assay*. Splenocytes (Sp) were isolated as previously described [9]. Briefly, a spleen was harvested from a naïve rat under anesthesia. The spleen was washed in 10 ml of PBS, homogenized using a gentleMACS Dissociator (Miltenyi Biotech). The cells were filtered through a 70 μm filter and centrifuged at 400*g* for 5 minutes. The cells were washed once again in PBS and resuspended in RPMI + 5% human AB serum. To evaluate the suppressive ability of Treg, MSC and Treg+MSC combination therapy, 2x10^5^ Sp were added to 96-well plates and activated with either lipopolysaccharide (LPS) or concanavalin A (ConA).

In the LPS activated plate, Treg (1:8), MSC (1:20) or combination of Treg (1:8) + MSC (1:20) were added to the wells, and the cell culture supernatant was collected after 24 hours. In the ConA activated plate, Treg (1:8), MSC (1:20) or combination of Treg (1:8) + MSC (1:20) were added to the wells, and the culture supernatant was collected after 72 hours. The samples were analyzed utilizing rat specific TNF-α and IFN-γ ELISA kit (BD Biosciences, San Jose, CA) per manufacturer’s protocol.

*Human PBMC activation assays*. Fresh human peripheral blood was collected from two healthy donors through approved IRB protocol HSC-MS-10-0190. Peripheral blood mononuclear cells (PBMC) were isolated using the same protocol as described above, using SepMate-50 tubes and Ficoll-Paque. The PBMC were then washed with PBS, counted and suspended in RPMI with 5% human AB serum. To evaluate the suppressive ability of Treg, MSC and Treg+MSC combination therapy, we performed a PBMC activation assay, in which 2x10^5^ PBMC were added to a 96-well plate and activated using CD3/28 biotinylated beads (Miltenyi Biotec) following the manufacture’s protocol [9]. Two separate in vitro experiments were performed using the two different PBMC donors on different dates. First, Treg (1:8), MSC (1:20) or combination of Treg (1:8) + MSC (1:20) were added to activated PBMC. The culture supernatant was collected at 72 hours. The samples were analyzed using human ELISA kits for TNF-α and IFN-γ (Biolegend, San Diego, CA) per the manufacturer’s protocol. In the second experiment, Treg were added at ratios of 1:4, 1:8, or 1:16 Treg:PBMC; MSC were added at a ratios of 1:10, 1:20, or 1:40 MSC:PBMC. Combination Treg (1:4) +MSC (1:10) was also assessed. The culture supernatant was collected at 72 hours. The samples were analyzed using human ELISA kits for TNF-α and IFN-γ (Biolegend, San Diego, CA) per the manufacturer’s protocol. In addition, these samples were also analyzed using human ELISA kits for AREG (R&D Systems, Minneapolis, MN) and PGE2 (Cayman Chemical Co., Ann Arbor, MI).

#### 3. In vivo experiments

*Controlled cortical impact model*. Animals were anesthetized with 4% isoflurane and oxygen in a vented chamber and then maintained at 2–3% isoflurane for the duration of the procedure. The animal was then secured on a stereotactic frame and the surgical site was prepped with alcohol and iodine solution. Subcutaneous 0.25% bupivacaine was administered prior to incision for local anesthesia. A midline cranial incision was made, and the right sided musculature and soft tissue were bluntly dissected away for exposure of the calvarium. A 7-mm diameter craniectomy was performed between the right coronal and lambdoid sutures. A controlled cortical impact device (Impact One Stereotaxic Impactor, Leica Microsystems, Buffalo Grove, IL) was utilized to administer a standardized and unilateral severe brain injury as previously described [21, 22]. Severe injury parameters included a depth of 3.1 mm, impact velocity of 5.6 m/s, and a dwell time of 150ms using a 6mm diameter impactor tip to the parietal association cortex. The craniectomy site was left open. Immediately after the injury, the incision was closed using sterile wound clips and animals were allowed to recover in newly cleaned micro-isolator cages provided by the University CLAMC. Sham injuries were performed by anesthetizing the animals, making the midline incision, and separating the skin, connective tissue and aponeurosis from the cranium. The incision was closed using sterile wound clips. There were no animal mortalities after injury.

**Fig 1:**
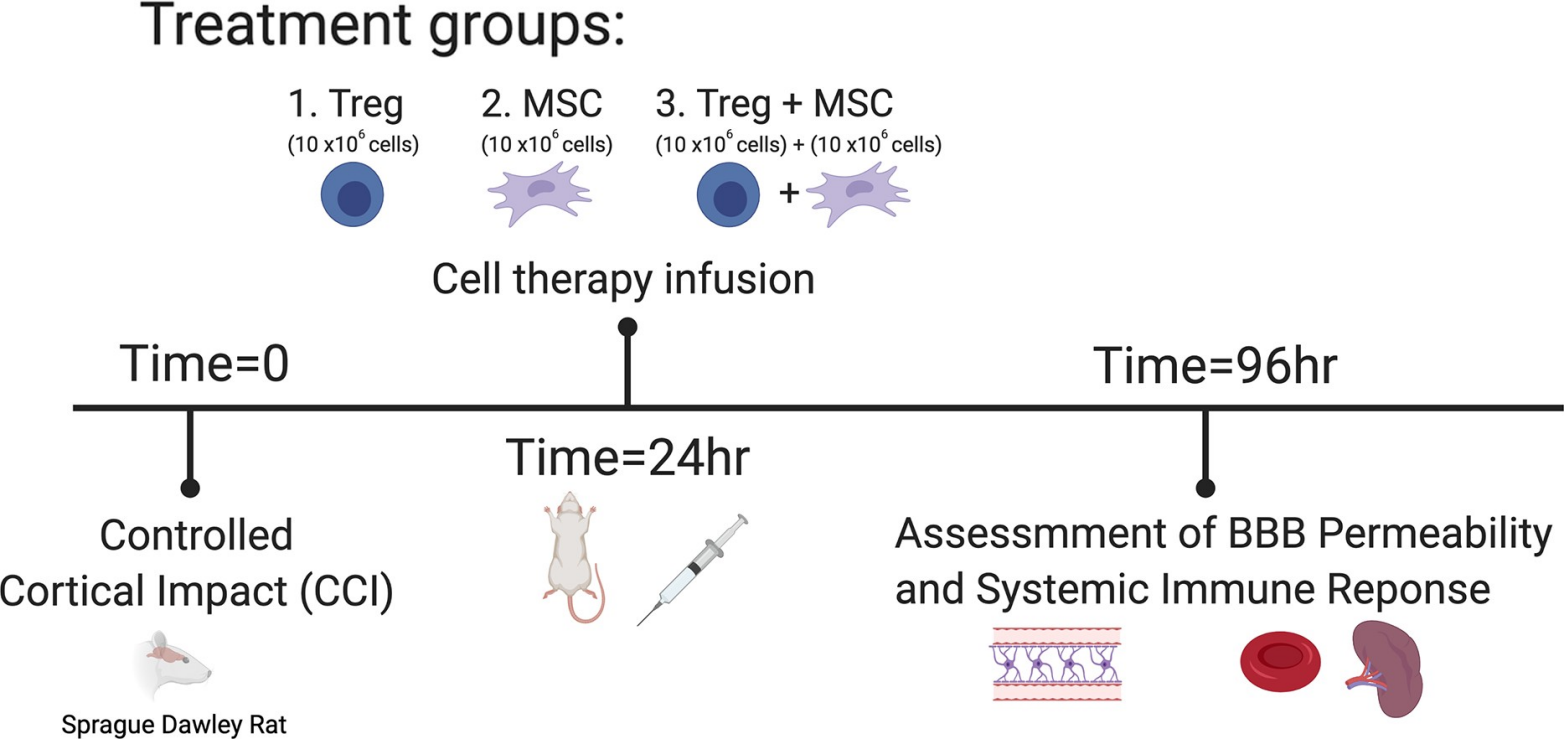
Experimental design and timing of treatment for experimental TBI model.

*Treg and MSC infusion*. After injury, animals were randomized to receive either 1) Treg alone (n = 6), 2) MSC alone (n = 6) or 3) Treg+MSC (n = 6) at 24 hours after injury (Fig 1). Treg and MSC were thawed and washed as described above. Treg and MSC were resuspended in 1mL of sterile PBS at a dose of 10x10^6^ cells/kg for each cell type, and cells were infused via tail vein injection at 24 hours after injury. No animal morbidity or mortality was observed after cell infusion.

*Experimental groups*. There were five experimental groups: Sham (n = 6), CCI (n = 6), Treg alone (n = 6), MSC alone (n = 6), and Treg+MSC (n = 6).

*Blood brain barrier permeability measurement*. At 96 hours after injury, BBB permeability was assed as previously described [22, 23]. Briefly, 1mg/kg Alexa 680 (Life Technologies, Thermo Fisher Scientific, Waltham, MA) was injected via tail vein thirty minutes before animals were euthanized. Following exsanguination, PBS and 4% paraformaldehyde perfusion, the brains were harvested. The brains were then sliced in 2-mm coronal sections and scanned for Alexa 680 using a LiCor Odyssey infrared laser scanner (LI-COR, Lincoln, NB). b. Quantitative measurements of dye extravasation were determined by thresholding to remove background followed by measuring the integrated density of fluorescent intensity of the slices by a blinded examiner using digital analysis (ImageJ). Specifically, the integrated density of each set of brain slices was measured using a uniform sized region of interest and restricted to a lower threshold of 257 and an upper threshold of 2827. This range was selected to exclude background fluorescence and the intense dye accumulating in the CCI lesion as previously published [23].

*Rat splenocyte and blood flow cytometry immunophenotyping*. Flow cytometry was performed on spleens and blood to characterize lymphoid and myeloid cell populations at 96 hours after injury as previously described [9]. The antibody panel consisted of the following cell surface markers: anti-CD3-FITC, anti-CD25-PE, anti-CD8a-PerCP, anti-CD11bc-PECy7, anti-RT1B-APC, anti-CD4-APCCy7, anti-CD45RA-V450. Myeloid cells were identified as CD11bc positive, CD3 negative, CD45RA negative cells. B cells were identified as CD45RA positive, CD3 negative, CD11bc negative cells. T cells were identified as CD3 positive, CD11bc negative, CD45RA negative cells. Further T cell subsets were identified using the CD4, CD8 and CD25 markers. At time of euthanasia, blood was collected via direct cardiac puncture in heparinized collection vials. 100 μL of blood was added directly to flow tubes and stained with the antibody panel. Spleens were harvested from animals at the time of euthanasia and immediately weighed. Splenocytes were isolated as described above and stained with the antibody cocktail. Data for the splenocyte and blood samples were acquired on a Gallios Flow Cytometer (Beckman Coulter). Subsequent flow cytometry analyses were completed using FlowJo vr10.6.1 (FlowJo, LLC).

*t-SNE visualization and quantification of rat splenocyte flow cytometry data*. t-SNE analysis was also performed using FlowJo vr10.6.1 and the same Flow Cytometry Standard (FCS) files used in the analyses above. Briefly, live cells were gated on all CD3+ cells. Within each sample, total CD3+ live cell events were randomly down-sampled to 3000 events, and analysis was run on equal numbers of events per sample. The individual sample files were concatenated to link them together into a single standard file. t-SNE was run using the FlowJo plugin. In each t-SNE figure, all samples and groups were derived from the same t-SNE run. We first analyzed the density plots of the individual groups and then generated antibody heat maps to visualize the fluorescent intensity of each antibody marker. Then, cell gates applied to visualize the different T cell phenotypes and number of cells within each gate were quantified to characterize changes in T cell populations after injury and treatment.

#### 4. Statistical analysis

All data were analyzed with GraphPad Prism (GraphPad Software, Inc., La Jolla, CA). Comparisons between means of sham/naïve and injured/activated controls within each group were analyzed using an unpaired T-test to demonstrate effectiveness of our in vivo experimental injury and in vitro activation models. Then, we compared means of the injury/activated control and treatment groups using ordinary one-way ANOVA with Dunnett’s multiple comparisons test. Values of p ≤ 0.05 were considered significant and indicated with (#) for p ≤ 0.05, (##) for p ≤ 0.01, (###) for p ≤ 0.001. Further post-hoc analysis to specifically compare the means of monotherapy and combination therapy groups were analyzed using one-way ANOVA with Sidak’s multiple comparisons test. All group data are presented as mean ± standard error. Values of p ≤ 0.05 were considered significant and indicated with (*) for p ≤ 0.05, (**) for p ≤ 0.01, (***) for p ≤ 0.001.

## Results

### Treg+MSC combination therapy does not improve restoration of vascular integrity compared to MSC or Treg monotherapies

**Fig 2:**
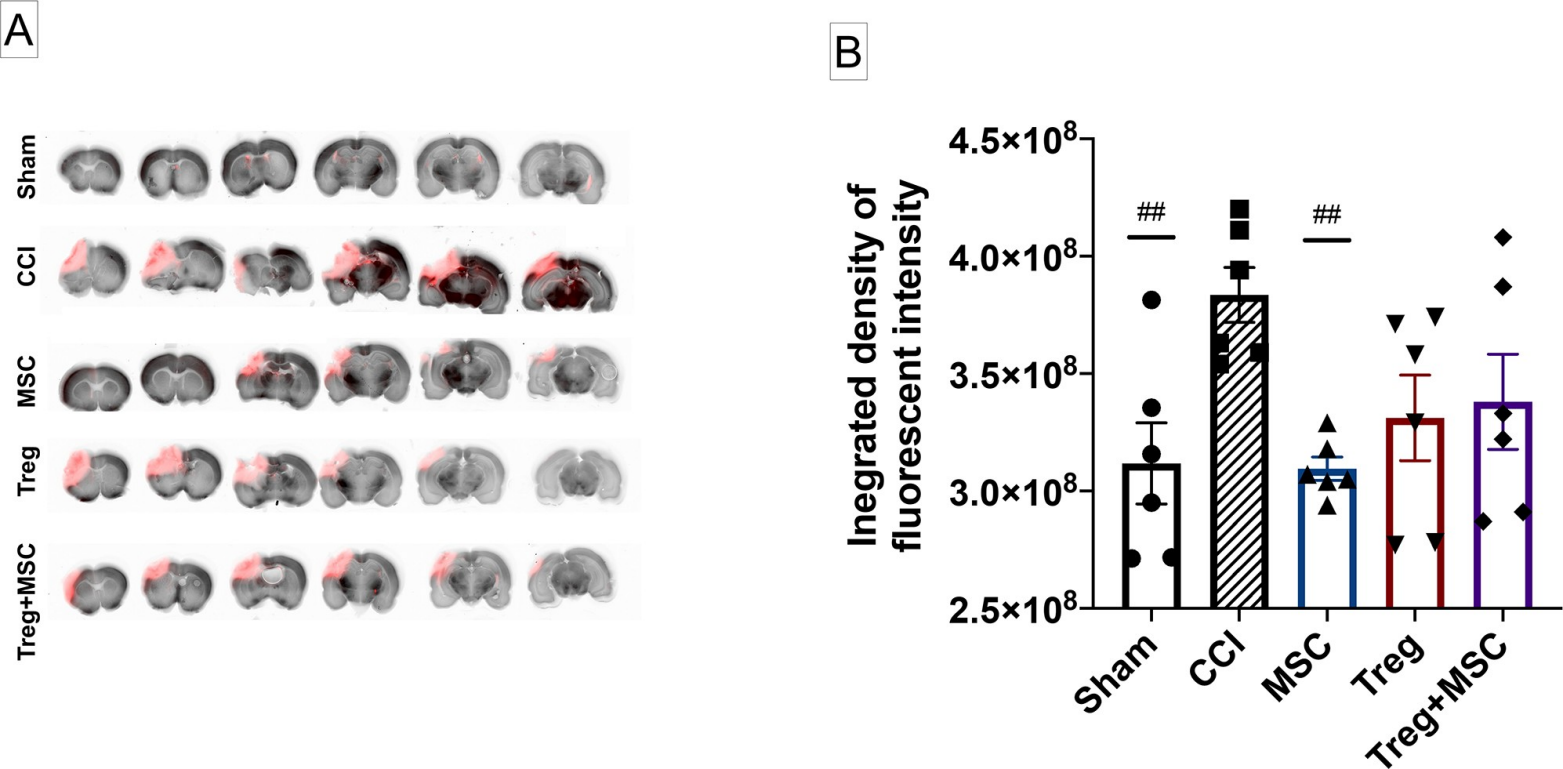
Assessment of blood brain barrier (BBB) permeability at 96 hours after CCI. **(A)**: Representative slices of the brain from forebrain to hindbrain, imaged using a LiCor Odyssey infrared scanner. Of note, the visual differences apparent between the groups do not directly correspond to quantitative changes in fluorescent intensity. **(B)**: Quantitative assessment of BBB permeability, measured using the integrated density of fluorescence between intensities of 257–2827 which excludes background fluorescence and the focal cortical lesion, demonstrates that the BBB remains disrupted after CCI at 96 hours compared to sham. MSC monotherapy significantly reduced BBB permeability, while Treg monotherapy (p = 0.059) and Treg+MSC (p = 0.11) trended towards improvement. There were no significant differences between treatment groups. N = 6. Values of p ≤ 0.05 were considered significant. Statistical significance between sham/treatment and CCI is indicated with (#) for p ≤ 0.05, (##) for p ≤ 0.01, (###) for p ≤ 0.001. Statistical significance between treatment groups is indicated with (*) for p ≤ 0.05, (**) for p ≤ 0.01, (***) for p ≤ 0.001. CCI, controlled cortical impact.

TBI causes disruption and increase in permeability of the BBB, which can worsen cerebral edema and negatively affect outcomes. We have previously demonstrated that MSC, but not Treg, can significantly restore BBB integrity after CCI [8, 9]. Therefore, we sought to determine whether Treg+MSC combination therapy would augment or inhibit BBB repair after CCI in comparison to MSC and Treg monotherapy. At 96 hours, we observed a persistent significant difference between sham and CCI when measuring the integrated density of dye accumulation in an intensity range that excluded background fluorescence and higher intensity staining that was generally localized to the lesion. This analysis is intended to preferentially analyze diffuse microvascular permeability associated with neuroinflammation rather than high-intensity vascular injuries that accumulate at the focal injury site. Although we have previously infused MSC at 72 hours, here we have shown that MSC monotherapy at 24 hours also significantly decreased BBB permeability compared to the injured control. Furthermore, Treg monotherapy (p = 0.059) and Treg+MSC (p = 0.11) trended towards improved BBB integrity (Fig 2). There were no significant differences between treatment groups, indicating that combination therapy did not negatively or positively contribute to BBB repair after injury.

### Treg+MSC combination therapy increases splenic CD4+CD25+ regulatory T lymphocytes in vivo, but not in comparison to Treg or MSC monotherapy

**Fig 3:**
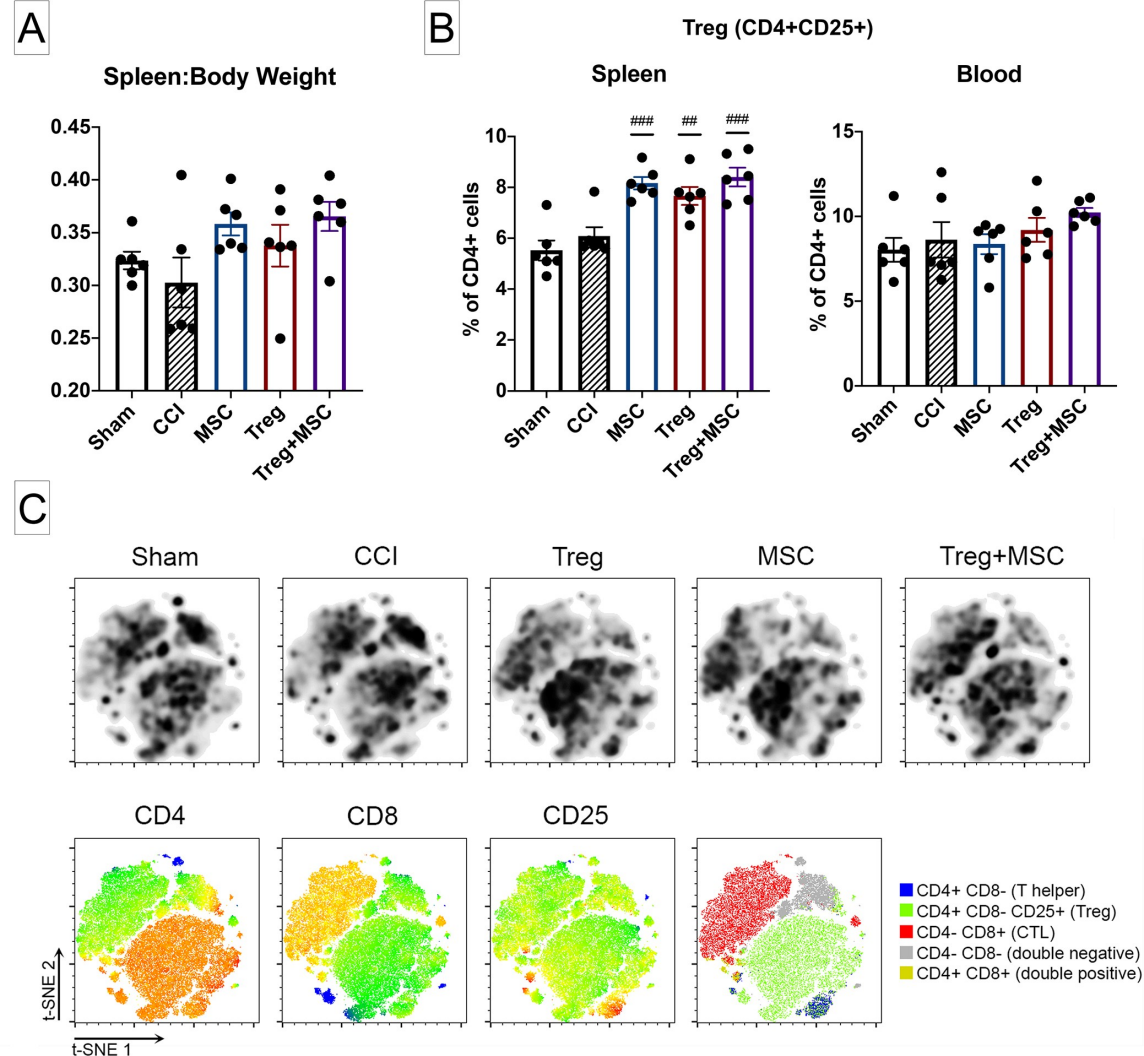
Changes in splenic weight and flow cytometric characterization of immune cells in the spleen and blood after CCI and treatment. **(A)**: Assessment of changes in the ratio of spleen to body weight at 96 hours after CCI. There were no significant differences between sham and CCI. However, there was a trend towards increased spleen:body weight after Treg+MSC (p = 0.055). **(B)**: Quantification of CD4+CD25+ rat Treg in the spleen and blood after CCI and treatment using flow cytometry logic-based gating. While there were no significant differences Sham and CCI in the spleen or blood, all three treatments increased the percentage of splenic Treg. There were no differences between treatments. **(C)**: t-SNE visualization of change in CD3+ T cell populations in the spleen after CCI and treatment. Density plots (top row) demonstrate distinct differences in cell clusters in the injured and treatment animals (black boxes). Analysis of antibody heat maps (bottom row) show that these boxed clusters are largely CD4+CD25+ cells. N = 6. Statistical significance between sham/treatment and CCI is indicated with (#) for p ≤ 0.05, (##) for p ≤ 0.01, (###) for p ≤ 0.001. Statistical significance between treatment groups is indicated with (*) for p ≤ 0.05, (**) for p ≤ 0.01, (***) for p ≤ 0.001. CCI, controlled cortical impact; Treg, regulatory T cell; t-SNE, t-distributed stochastic neighbor embedding.

We have previously hypothesized that the interaction between cell therapy and the host immune system likely confers the therapeutic benefit in TBI. Here, we evaluated the effects of Treg, MSC, and Treg+MSC combination therapy on the spleen:body mass ratio and spleen and blood immune cells populations at 96 hours after injury. Previous work has demonstrated that cell therapy can preserve splenic mass at 72 hours after CCI [11]. Although there was no significant difference between sham and CCI, there was a trend towards an increased ratio of spleen:body mass in the Treg+MSC group (p = 0.055) (Fig 3A).

In addition, we evaluated the changes in splenocyte and blood immune cell populations after CCI and treatment. All three treatments increased the percentage of Treg (CD4+CD25+CD8-) in the splenocyte populations; however, no similar treatment effect was seen in the blood (Fig 3B). The only other differences observed at 96 hours after injury were an increase in myeloid cells (CD11+) in the spleen and blood and a decrease in B cells (CD45RA+) in the blood in the injured versus sham controls (S1 Fig).

We then performed t-SNE on the CD3+ cells from each sample. t-SNE visualization of the splenic T cell populations also demonstrated an increase in density and intensity of CD25+ cells in the treatment groups compared to both sham and injured controls (Fig 3C). We further quantified these changes by applying cell gating strategies to characterize changes in T cell populations after injury and treatment. Similar to our conventional flow cytometry analysis, there were no differences in T cell populations between sham and CCI. However, tSNE quantification revealed that only Treg+MSC combination therapy had a statistically significant increase in total percentage of Treg (all CD3+ cells) and percentage of Treg within the CD4+CD8- gate (S2 Fig). In addition, we utilize t-SNE to identify cell populations that we might otherwise ignore using our conventional flow cytometry gating and analysis. Here, there are clear differences in the CD4-CD8- double negative T cell population after all treatments. Quantification of cells within this gate demonstrates a statistically significant decrease in double negative T cells after all three treatments (S2 Fig).

### Immunomodulatory effects of Treg+MSC combination therapy compared to monotherapy on activated rat splenocytes in vitro

**Fig 4:**
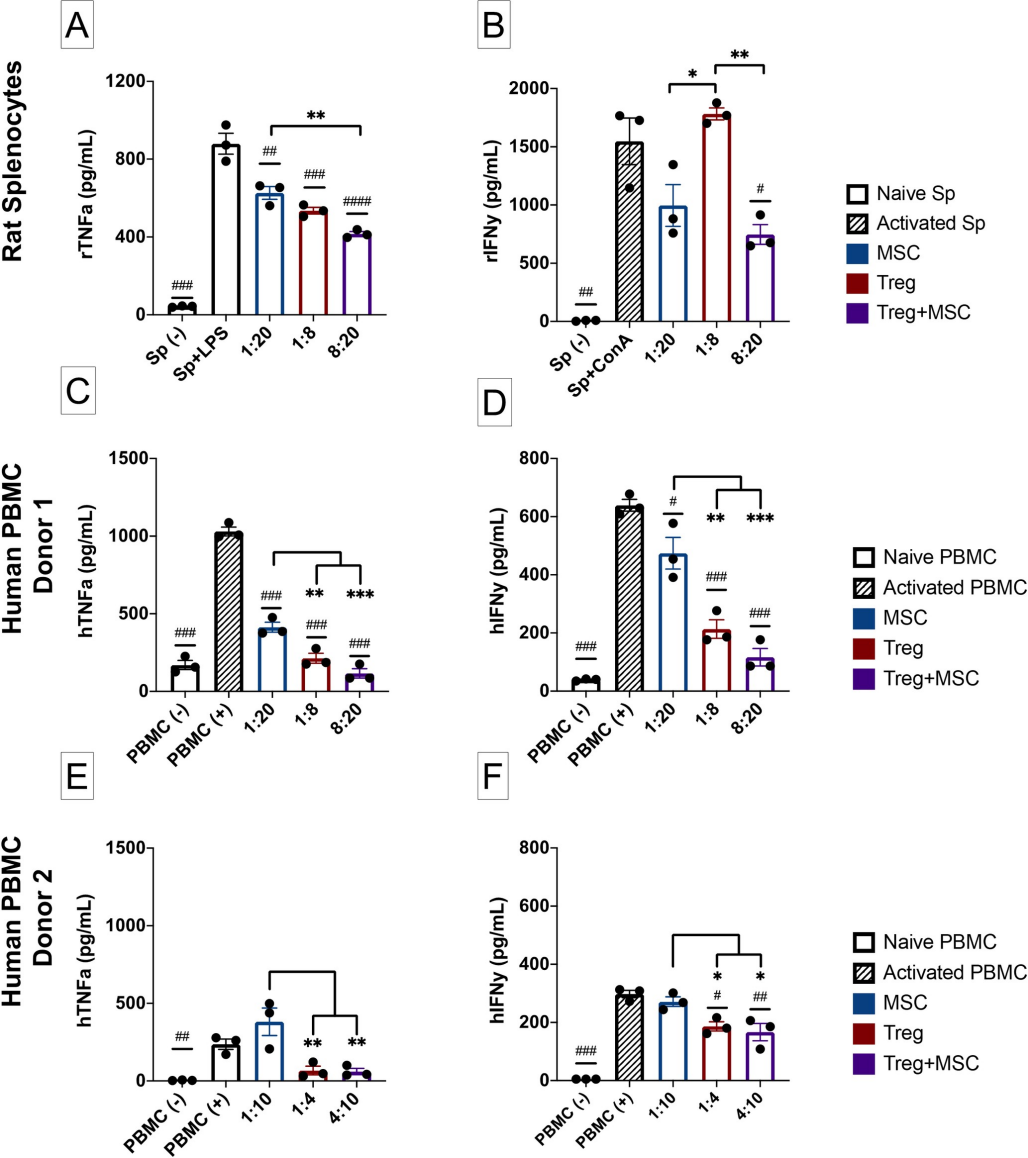
Characterization of immune suppressive potential of MSC, Treg, and Treg+MSC on activated rat splenocytes and human PBMC in vitro. **(A)**: TNF-a production (ELISA) by rat splenocytes after LPS stimulation. **(B)**: IFNy production (ELISA) by rat splenocytes after ConA stimulation. **(C-D)**: Pro-inflammatory cytokine production, TNFa (C) and IFNy (D) by activated human Donor 1 PBMC after anti-CD3/CD28 bead stimulation (ELISA). **(E-F)**: Pro-inflammatory cytokine production, TNFa (E) and IFNy (F) by activated human Donor 2 PBMC after anti-CD3/CD28 bead stimulation (ELISA). All samples run in triplicate. Statistical significance between naive/treatment and activated control is indicated with (#) for p ≤ 0.05, (##) for p ≤ 0.01, (###) for p ≤ 0.001. Statistical significance between treatment groups is indicated with (*) for p ≤ 0.05, (**) for p ≤ 0.01, (***) for p ≤ 0.001. Treg, regulatory T cell; MSC, mesenchymal stromal cell; PBMC, peripheral blood mononuclear cells; TNFa, tumor necrosis factor alpha; IFNy, interferon gamma; ELISA, enzyme-linked immunosorbent assay.

The systemic immune response to TBI has significant effects on secondary injury and progression of subacute and chronic neuroinflammation, and we have previously demonstrated that both Treg and MSC can modulate the innate and adaptive immune responses of activated rat splenocytes in vitro [8, 9]. However, the effects of combination therapy on rat immune responses is unknown. Of note, in this in vitro experiment, we used lower doses of Treg than previously reported in order to better assess the potency of combination therapy. After LPS stimulation, Treg and MSC monotherapies and Treg+MSC combination therapy all significantly reduced TNFa production. Furthermore, there was a significant decrease in TNFa after combination therapy compared to MSC monotherapy (Fig 4A). After ConA stimulation, only Treg+MSC reduced IFNy in comparison to the activated control. In addition, there were significant decreases in IFNy production in both the MSC monotherapy and Treg+MSC combination therapy compared to Treg monotherapy (Fig 4B). Thus, it appears that combination therapy may confer the ability to better attenuate both the innate and adaptive immune responses of activated rat splenocytes in comparison to either Treg or MSC monotherapies.

### Immunomodulatory effects of Treg+MSC combination therapy compared to monotherapy on activated human PBMC in vitro

While we investigated the effects of cell therapy on rat immune cells to correlate our in vivo findings, we also sought to examine the effects of combination therapy on activated human PBMC as another marker of potency and efficacy. Using two different donor PBMC, we stimulated PBMC with anti-CD3 and CD28 beads in order to induce an effector T cell response. In the first assay (PBMC Donor 1), all three treatments significantly reduced both TNFa and IFNy production compared to the activated control (Fig 4C and 4D). In addition, Treg monotherapy and combination therapy significantly reduced the pro-inflammatory cytokine production compared to BM-MSC monotherapy.

In a second assay (PBMC Donor 2), we observed decreased attenuation of pro-inflammatory cytokine production by all treatments; however, the Donor 2 PBMC also demonstrated a decreased pro-inflammatory response compared to Donor 1. Treg monotherapy (p = 0.11) and combination therapy (p = 0.092) demonstrated a trend towards decreasing TNFa production, while MSC monotherapy actually increased cytokine production. With respect to IFNy, both Treg monotherapy and combination therapy significantly inhibited cytokine production, while MSC monotherapy did not. Interestingly, although treatment potency between the two assays varied, the relationship between the three therapies remained consistent: Treg monotherapy and combination therapy both demonstrated significantly improved attenuation of both pro-inflammatory cytokines compared to MSC monotherapy (Fig 4E and 4F). These findings highlight the potential variability of such in vitro assays, including donor PBMC activation and potential differences in effect of cell therapy on host PBMC. Furthermore, these data also related that combination therapy may provide benefit over MSC monotherapy with respect to pro-inflammatory cytokine production by activated immune cells.

### Differences in PGE2 and AREG production after Treg, MSC and Treg+MSC combination therapy

In addition to measurement of cytokine inhibition, we also measured secretion of two potential anti-inflammatory mediators, PGE2 and AREG, to examine the effects of Treg and MSC monotherapy and the potential effects of combination therapy on such. While we have previously demonstrated that PGE2 correlates with therapeutic benefit of MSC, we have not examined PGE2 production after Treg treatment or the effects of combination therapy on PGE2 production [8].

**Fig 5:**
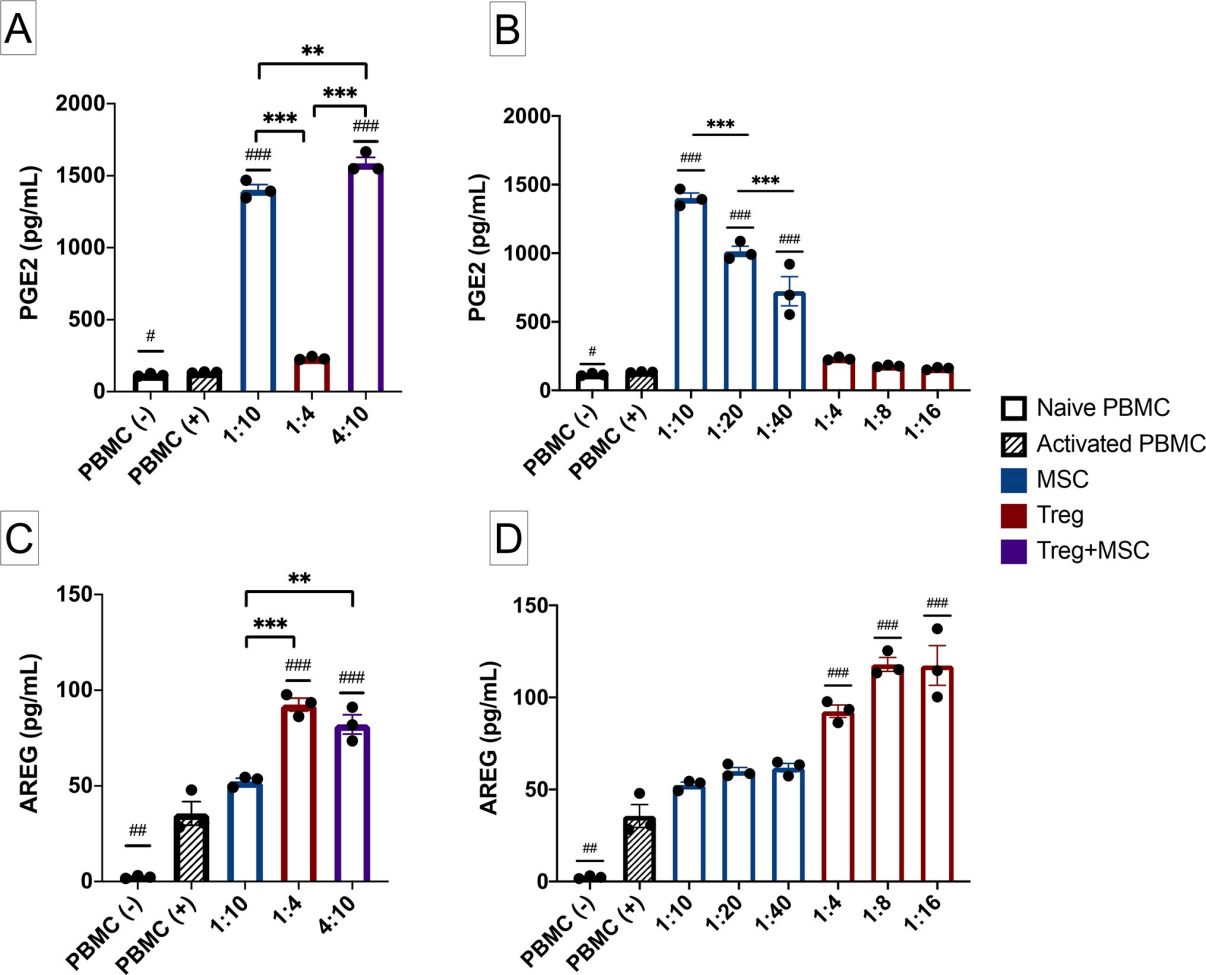
PGE2 and AREG production by Treg, MSC and Treg+MSC in vitro. **(A)**: PGE2 production by activated human PBMC and MSC, Treg, and Treg+MSC treatments (ELISA). **(B)**: PGE2 production by activated human PBMC and various ratios of MSC and Treg treatments (ELISA). **(C)**: AREG production by activated human PBMC and MSC, Treg, and Treg+MSC treatments (ELISA). **(D)**: AREG production by activated human PBMC and various ratios of MSC and Treg treatments (ELISA). All samples run in triplicate. Statistical significance between naive/treatment and activated control is indicated with (#) for p ≤ 0.05, (##) for p ≤ 0.01, (###) for p ≤ 0.001. Statistical significance between treatment groups is indicated with (*) for p ≤ 0.05, (**) for p ≤ 0.01, (***) for p ≤ 0.001. PGE2, prostaglandin E2; AREG, amphiregulin; PBMC, peripheral blood mononuclear cells; Treg, regulatory T cell; MSC, mesenchymal stromal cell; ELISA, enzyme-linked immunosorbent assay.

MSC therapy, but not Treg monotherapy, significantly increased PGE2 levels more than activated PBMC (Fig 5A and 5B); furthermore, there is a clear dose response of PGE2 production and increasing MSC doses. In addition, Treg+MSC combination therapy increased PGE2 production compared to the respective MSC monotherapy (5a). However, only Treg monotherapy and Treg+MSC combination therapy increase AREG production compared to activated PBMC (Fig 5C). No such dose response was observed with respect to AREG production and Treg dose (Fig 5D). Altogether, these data demonstrate that MSC and Treg may possess unique anti-inflammatory mechanisms: MSC upregulate PGE2 production, while Treg upregulate the production of AREG.

## Discussion

In this study, we have compared the effects of Treg+MSC combination therapy to Treg and MSC monotherapies. Specifically, we tested the ability of Treg+MSC combination therapy to decrease BBB disruption after TBI in an in vivo rat model, as well as the ability to inhibit inflammatory responses in vitro. To our knowledge, this is one of few studies to examine the effects of human Treg and MSC combination therapy [12].

Damage to the BBB is common in severe TBI, and prolonged disruption can worsen cerebral edema, allow influx of pro-inflammatory mediators, and further aggravate secondary brain injury [24]. Here, we demonstrate that significant BBB disruption remains at 96 hours after CCI and that MSC monotherapy at 24 hours significantly reduced BBB permeability by certain measurements, supporting an improvement in vascular integrity outside of the focal injury. However, we did not observe any treatment advantage of Treg+MSC combination therapy. While Treg monotherapy and Treg+MSC combination therapy trended towards decreasing BBB permeability in comparison to injured controls (p = 0.08 and p = 0.15, respectively), we concluded that the number of animals needed to reach significance would not justify the outcome. Future investigations should examine the relationship between timing of therapy (e.g. cell infusions at 24 versus 72 hours) and the effects of multiple or staggered infusions on the ability of cell therapy to restore BBB integrity after TBI [5].

TBI also alters the systemic immune system and, specifically, causes deficits in the adaptive immune response, including decreases in splenic Treg populations [25–27]. Previously we have demonstrated that human Treg monotherapy increased the percentage of rat Treg populations after injury [9]. In this experiment, all three treatment regimens increased the percentage of Treg cells in the spleen, a finding which was supported by t-SNE visualization of changes in splenic CD3+ cell populations. Interestingly, quantification of T cell populations from our t-SNE analysis revealed that only Treg+MSC combination therapy significantly increased the percentage of Treg compared to the injured controls. As we have previously posited, the interaction between cell therapy and the endogenous immune system, specifically Treg, may an important mechanism for decreasing the harmful neuroinflammatory response to TBI [11]. Similar to our BBB findings, we did not observe any significant differences between Treg+MSC and Treg or MSC monotherapies. By what mechanism human-derived Treg and MSC can directly augment the splenic Treg response, and whether Treg and MSC utilize differing mechanisms, deserves further study. Furthermore, while the rat splenic Treg populations were significantly increased compared to the injured control, these values were not outside the normal limit of frequencies of Treg within the CD4 compartment in rats [28]. Therefore, whether this small, albeit statistically significant, increase in splenic Treg populations has any effect on outcomes deserves further study.

In addition, our t-SNE analysis allowed us to visualize changes in another cell population, the CD4-D8- double negative T cells (DNT), that we did not account for in our traditional flow cytometry analysis. Specifically, t-SNE visual analysis and quantification demonstrate that all three treatments significantly decreased the number of DNT compared to the injured control. The nature and role of DNT in inflammatory responses is complex, as they have been shown to have pro-inflammatory as well as tolerance promoting and immunosuppressive functions [29]. Interestingly, in a stroke model, Meng et al. recently demonstrated that DNT infiltrated the central nervous system (CNS), produced TNFa, and amplified pro-inflammatory microglia after stroke, ultimately enhancing neuroinflammation and further brain injury [30]. These data demonstrate that therapeutics that attenuate the DNT response may decrease microglia-mediated neuroinflammation, a common pathologic consequence shared by stroke and TBI [3]. Therefore, the attenuation of splenic DNT provides another example of how both Treg and MSC cell therapies may alter the systemic immune response in a neuro-protective manner.

Our in vitro data demonstrated that both Treg and MSC monotherapies can effectively attenuate pro-inflammatory cytokine production by activated immune cells, as well as the potential increased potency of Treg+MSC combination therapy compared to both monotherapies. We first examined the effect of these cell therapies on activated rat splenocytes. Of note, we used a lower Treg dose than in our previous study to better assess the potential advantage of Treg+MSC combination therapy. While all three treatments reduced TNFa production, only Treg+MSC combination therapy reduced IFNy production, indicating that combination therapy may confer an advantage over monotherapy in suppressing adaptive immune responses in rat. We have previously demonstrated CCI causes a significant increase in the adaptive immune response, and decrease in the innate immune response, of rat splenocytes [9]. Our data here suggest that Treg+MSC therapy may improve the ability to attenuate adaptive immune responses compared to monotherapy. Further investigation is warranted to study the specific effects of combination therapy on long-term adaptive immune responses, both systemic and within the CNS, after TBI.

We also examined the effects of combination therapy on activated human PBMC. While there was variability in the activation response by the two PBMC donors, these data demonstrate the immunosuppressive potential of all three treatment regimens. Furthermore, there was a consistent decrease in both TNFa and IFNy by Treg monotherapy and Treg+MSC combination therapy in comparison to MSC monotherapy, suggesting that Treg may be the primary driver of immune suppression of combination therapy in the context of this assay. This stands to reason as, here, PBMC specifically activated using anti-CD3 and CD28 beads in order to induce an effector T cell response.

**Fig 6:**
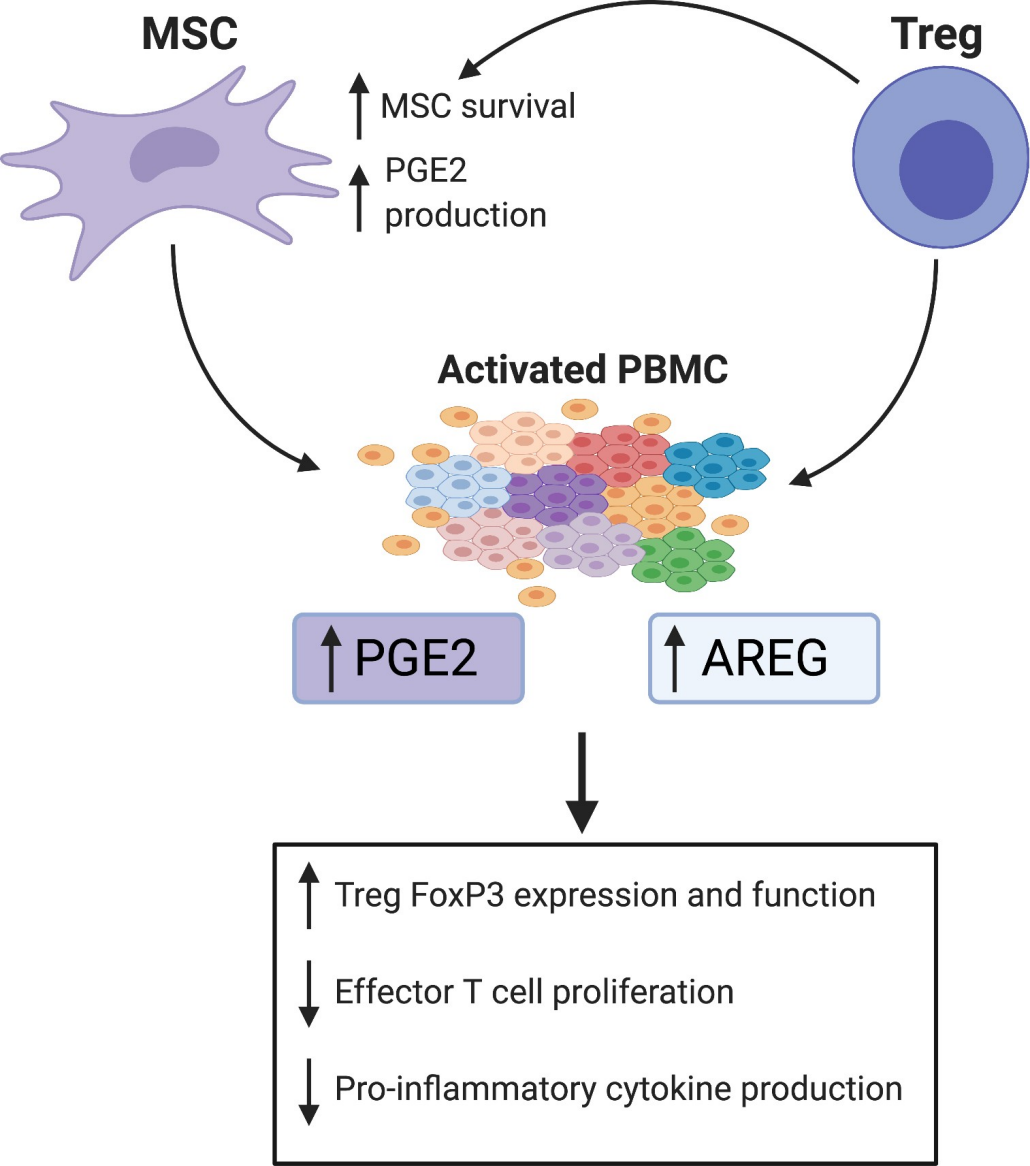
Potential mechanisms of the effects of PGE2 and AREG mediated therapeutic benefit of Treg+MSC combination therapy. PGE2 and AREG have been demonstrated as key mediators of immunomodulation, and our data suggests that MSC and Treg lead to significant increases in PGE2 and AREG production, respectively, in our activated PBMC co-culture. MSC are known to produce PGE2, likely a key determinant of their therapeutic potency [8]. PGE2 has many downstream effects, including polarization of anti-inflammatory, AREG-producing macrophages, induction of FoxP3 expression on Treg and augmentation of suppressive potential, and decreasing effector T cell proliferation and cytokine production [17, 18, 32]. AREG produced by other immune cells can augment Treg suppressive functions. Furthermore, Treg produce AREG at sites of injury, which can promote immune suppression and tissue healing [19]. In addition, Treg also may increase MSC survival in vivo and, as we have demonstrated, increase MSC production of PGE2 [16]. Thus, via augmented production of both PGE2 and AREG compared to Treg or MSC monotherapies, Treg+MSC combination therapy may afford enhanced immunomodulatory potency.

Finally, we examined differences in production of two known inflammatory mediators, PGE2 and AREG, by the various cell therapy treatments. These data demonstrate that Treg and MSC may utilize differing mechanisms to confer their therapeutic, anti-inflammatory benefits: MSC upregulated PGE2 production, while Treg therapy significantly increased AREG production. Furthermore, combination therapy led to significant increases in production of both PGE2 and AREG, which may confer a unique therapeutic benefit over both monotherapies. The augmented production of and potential therapeutic benefit of PGE2 by MSC has been extensively reviewed, and previous work in our lab has shown therapeutic potency of individual MSC preparations correlated with secretion of PGE2 [8, 18, 31]. Furthermore, PGE2 has been shown to induce forkhead box protein P3 (FoxP3) expression and augment Treg suppressive function in cultured blood CD4+ cells [31, 32]. AREG is a potent anti-inflammatory mediator produced and utilized by Treg, as well as many other immune cell types to promote tissue repair and suppress inflammation; however, to what extent AREG is critical or necessary for Treg function is still under investigation [19, 33, 34]. Interestingly, Ito et al. recently demonstrated that AREG produced by brain-derived Treg was critical for regulation of astrogliosis and neurologic recovery in a murine stroke model [35]. In addition, MSC-derived PGE2 may contribute to enhanced Treg function via polarization of AREG-secreting macrophages [17]. Therefore, while the breadth of mechanisms that MSC and Treg employ to modulate the immune system is beyond the scope of this discussion, PGE2 and AREG appear intricately linked with respect to both MSC and Treg function (Fig 6). However, to our knowledge, there have been no studies to directly compare the effects of Treg, MSC or Treg+MSC combination therapy on PGE2 or AREG production. Our data show that combination therapy may provide increase potency and therapeutic benefit over both Treg and MSC monotherapies, as only combination therapy resulted in higher levels of both AREG and PGE2.

Our study has several limitations. First, we analyzed the effects of TBI and cell therapy on BBB integrity using a single method. Future studies should include an examination of molecular markers of BBB integrity, such as tight junction proteins. In addition, our in vitro data analyzed the effects of these cell therapies on just two human donor PBMC populations, using ELISA to assess cytokine production. Future studies will aim to elucidate the effects of Treg+MSC combination therapy on a more granular, single-cell level and should certainly incorporate more human donors to better assess translatable potential. Finally, we did not correct for overall cell numbers in our in vivo or in vitro experiments; thus, the effects of combination therapy could simply be due to in an increase in the number of anti-inflammatory cells. Future studies will include appropriate dose controls of monotherapies.

In addition, this study highlights several key questions that are fundamental to testing Treg and MSC therapies. We still lack knowledge regarding the effects of infusion human-derived cells into immunocompetent rodents. There are very likely xenogeneic effects associated that we have not appreciated, which may significantly alter our ability to effectively analyze the therapeutic potential of these cell therapies [36]. Finally, does combination therapy effect the survival of Treg or MSC in vivo? Others have demonstrated that Treg may survive for prolonged periods of time after infusion in humans [37]. However, the data in rats is lacking and it is likely that a xenogenic immune response may severely limit the efficacy of human cells in an immunocompetent rat model. With respect to MSC survival, we and others have shown that the vast majority of MSC become trapped in the pulmonary microvasculature and do not reach the “target” organs [38].

## Conclusion

While our in vitro data demonstrates that combination therapy may augment therapeutic potency and immunosuppressive potential, Treg+MSC combination did not significantly improve BBB permeability or augment the endogenous immune response in vivo. There are many factors that may contribute to the efficacy of combination therapy for TBI, specifically timing of infusion and dosing. We believe that further studies are necessary to determine if combination therapy portends any true benefit in comparison to monotherapy.

## Supporting information

S1 Fig Flow cytometry characterization of changes in immune cell populations in the spleen and blood after CCI and treatment. Quantitative analysis of immune cell populations using flow cytometry logic-based gating. At 96 hours after injury, there were no differences between sham or treatment and CCI in the percentage of CD3+ T cells, CD4+ T cells, CD8+ T cells, or the ratio of CD4:CD8 T cells. There was a significant increase in CD11+ myeloid cells populations in the spleen and blood after CCI, but differences between CCI and any treatment group. Furthermore, there was a decrease in the percentage of CD45RA+ B cells in the blood, but no the spleen, in the CCI compared to sham. N = 6. Values of p ≤ 0.05 were considered significant. Statistical significance between sham/treatment and CCI is indicated with (#) for p ≤ 0.05, (##) for p ≤ 0.01, (###) for p ≤ 0.001. Statistical significance between treatment groups is indicated with (*) for p ≤ 0.05, (**) for p ≤ 0.01, (***) for p ≤ 0.001. CCI, controlled cortical impact.
(TIF)

S2 Fig Quantification of T cell populations in the spleen based upon distributions visualized using t-SNE. The concatenated data set composed of equally downsampled CD3-gated events from each animal was reanalyzed using the same logic gates used to generate Fig 3C. The frequency of CD4+CD25+ Tregs is presented as both a percentage of CD3+ cells and as a percentage of CD4+CD8- T helper cells (top row, left and right panel, respectively). Values of p ≤ 0.05 were considered significant. Statistical significance between sham/treatment and CCI is indicated with (#) for p ≤ 0.05, (##) for p ≤ 0.01, (###) for p ≤ 0.001.
(TIF)

S1 Table Multicolor flow cytometry rat immune cell panel. A table summary of the specific multicolor fluorescent antibody panel used to evaluate changes in rat immune cell populations for reference.
(DOCX)

S1 Data (ZIP)
