# Supplementary figures and images for: Human-derived Treg and MSC combination therapy may augment immunosuppressive potency in vitro, but did not improve blood brain barrier integrity in an experimental rat traumatic brain injury model
Source: PLoS One. 2021 May 26;16(5):e0251601. doi: 10.1371/journal.pone.0251601 (PMC8153465; doi:10.1371/journal.pone.0251601)

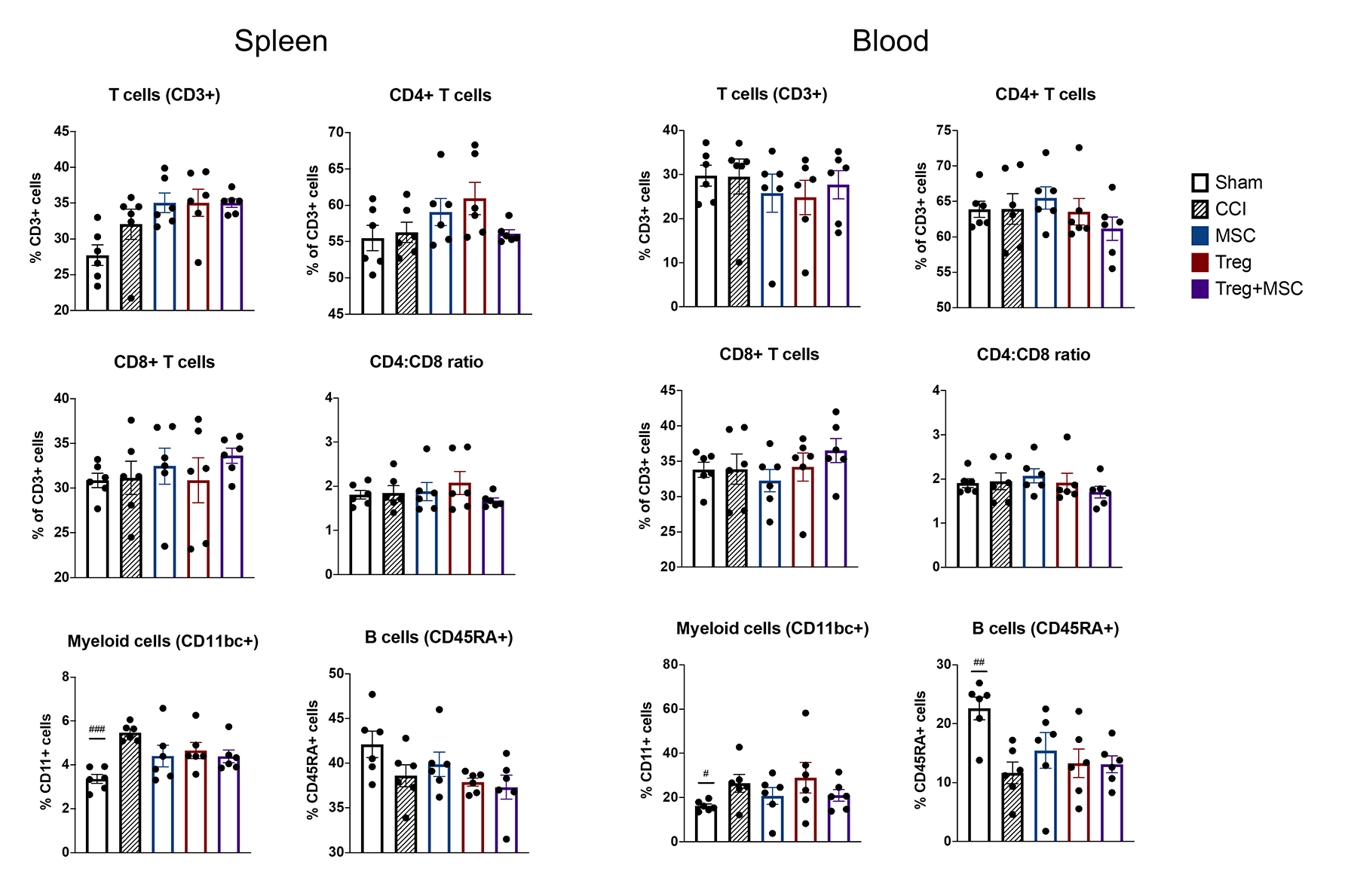

Supplement: S1 Fig — Quantitative analysis of immune cell populations using flow cytometry logic-based gating. At 96 hours after injury, there were no differences between sham or treatment and CCI in the percentage of CD3+ T cells, CD4+ T cells, CD8+ T cells, or the ratio of CD4:CD8 T cells. There was a significant increase in CD11+ myeloid cells populations in the spleen and blood after CCI, but differences between CCI and any treatment group. Furthermore, there was a decrease in the percentage of CD45RA+ B cells in the blood, but no the spleen, in the CCI compared to sham. N = 6. Values of p ≤ 0.05 were considered significant. Statistical significance between sham/treatment and CCI is indicated with (#) for p ≤ 0.05, (##) for p ≤ 0.01, (###) for p ≤ 0.001. Statistical significance between treatment groups is indicated with (*) for p ≤ 0.05, (**) for p ≤ 0.01, (***) for p ≤ 0.001. CCI, controlled cortical impact. (TIF) [file pone.0251601.s001.tif]

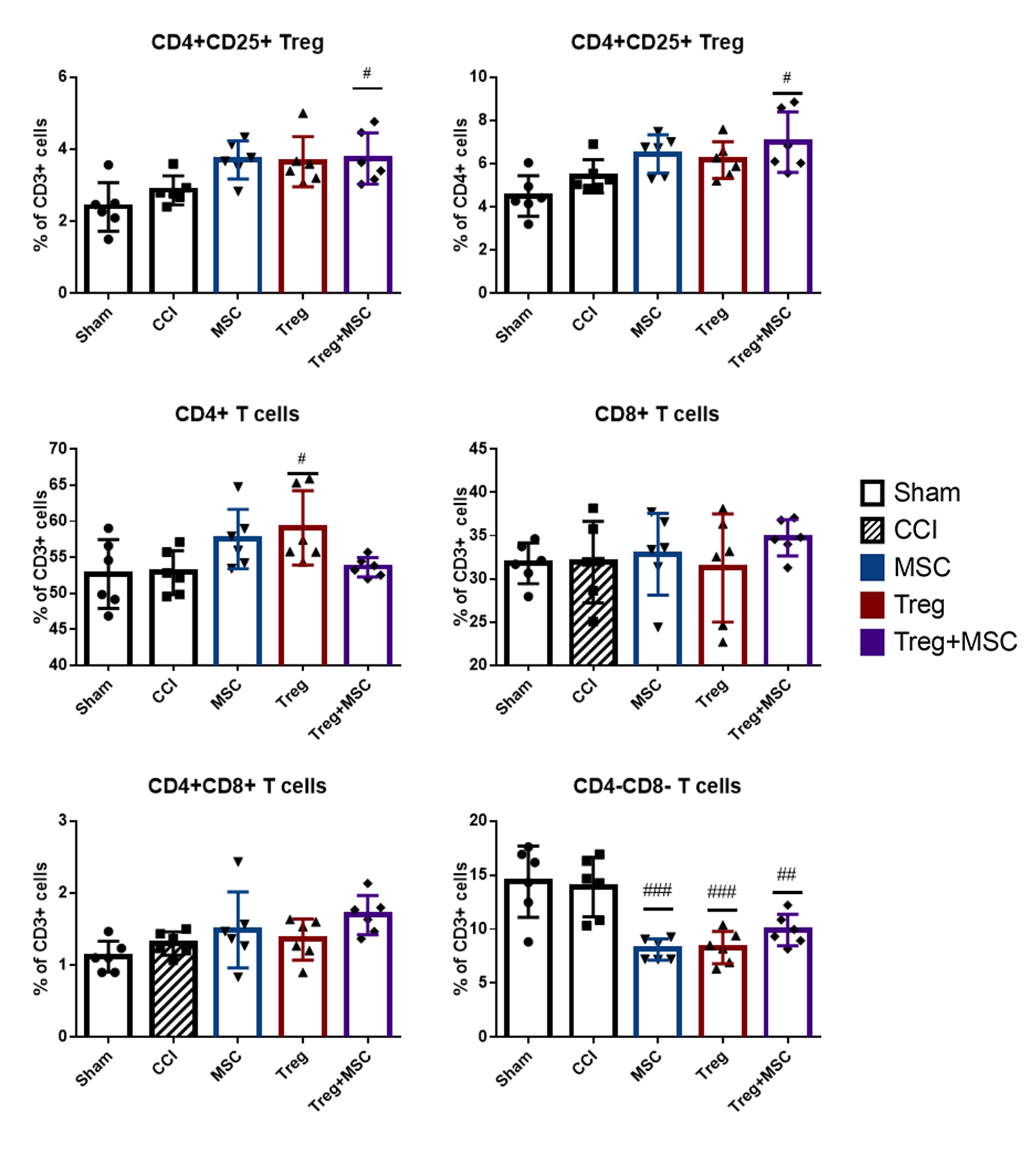

Supplement: S2 Fig — The concatenated data set composed of equally downsampled CD3-gated events from each animal was reanalyzed using the same logic gates used to generate Fig 3C. The frequency of CD4+CD25+ Tregs is presented as both a percentage of CD3+ cells and as a percentage of CD4+CD8- T helper cells (top row, left and right panel, respectively). Values of p ≤ 0.05 were considered significant. Statistical significance between sham/treatment and CCI is indicated with (#) for p ≤ 0.05, (##) for p ≤ 0.01, (###) for p ≤ 0.001. (TIF) [file pone.0251601.s002.tif]

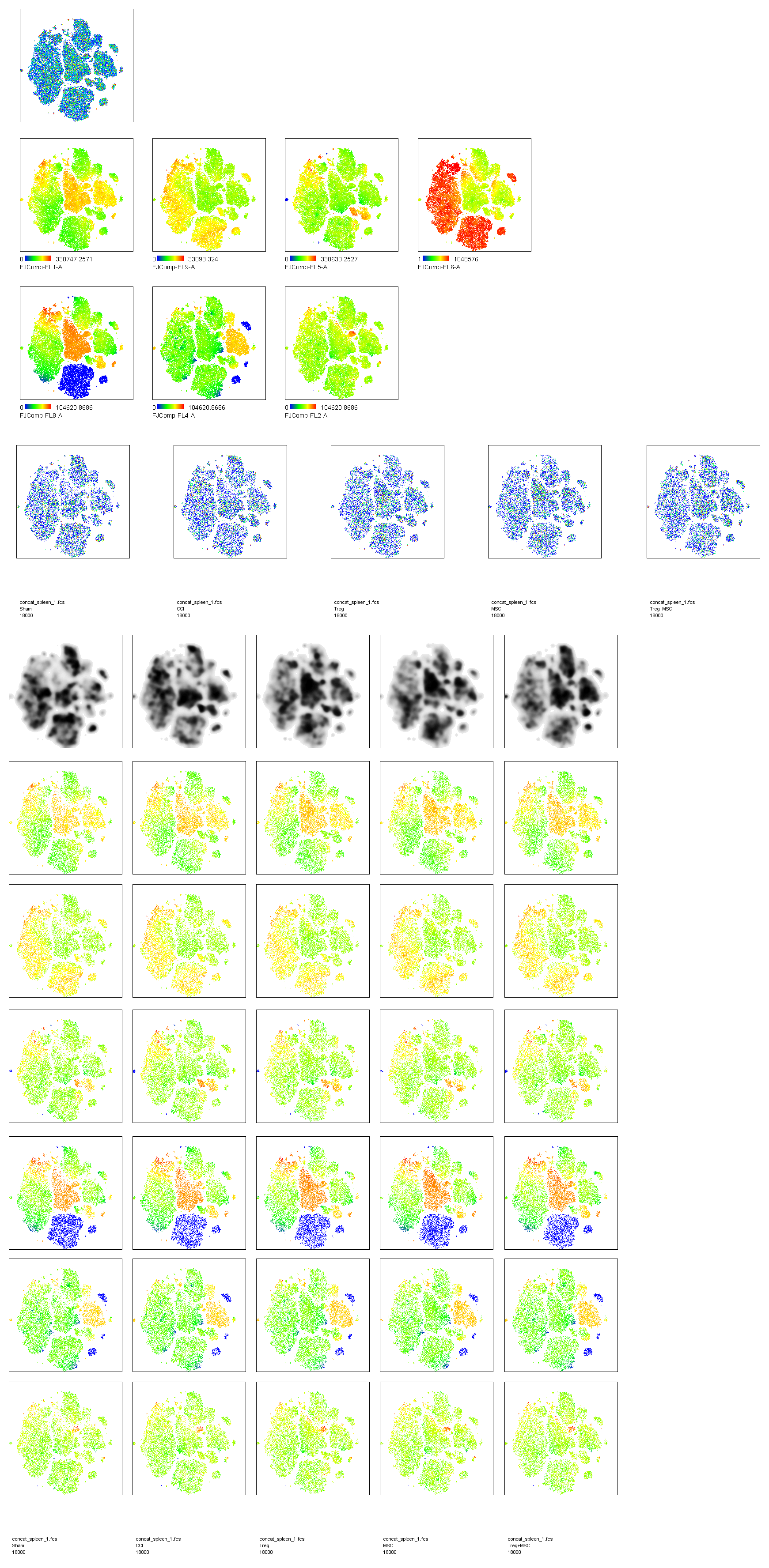

Supplement: S1 Data — (ZIP) [file pone.0251601.s004.zip › in vivo/11-26-20 96hr blood+spleen stats/treg+MSC 96hr 11-26-19 spleen tsne 5-29-20.png]

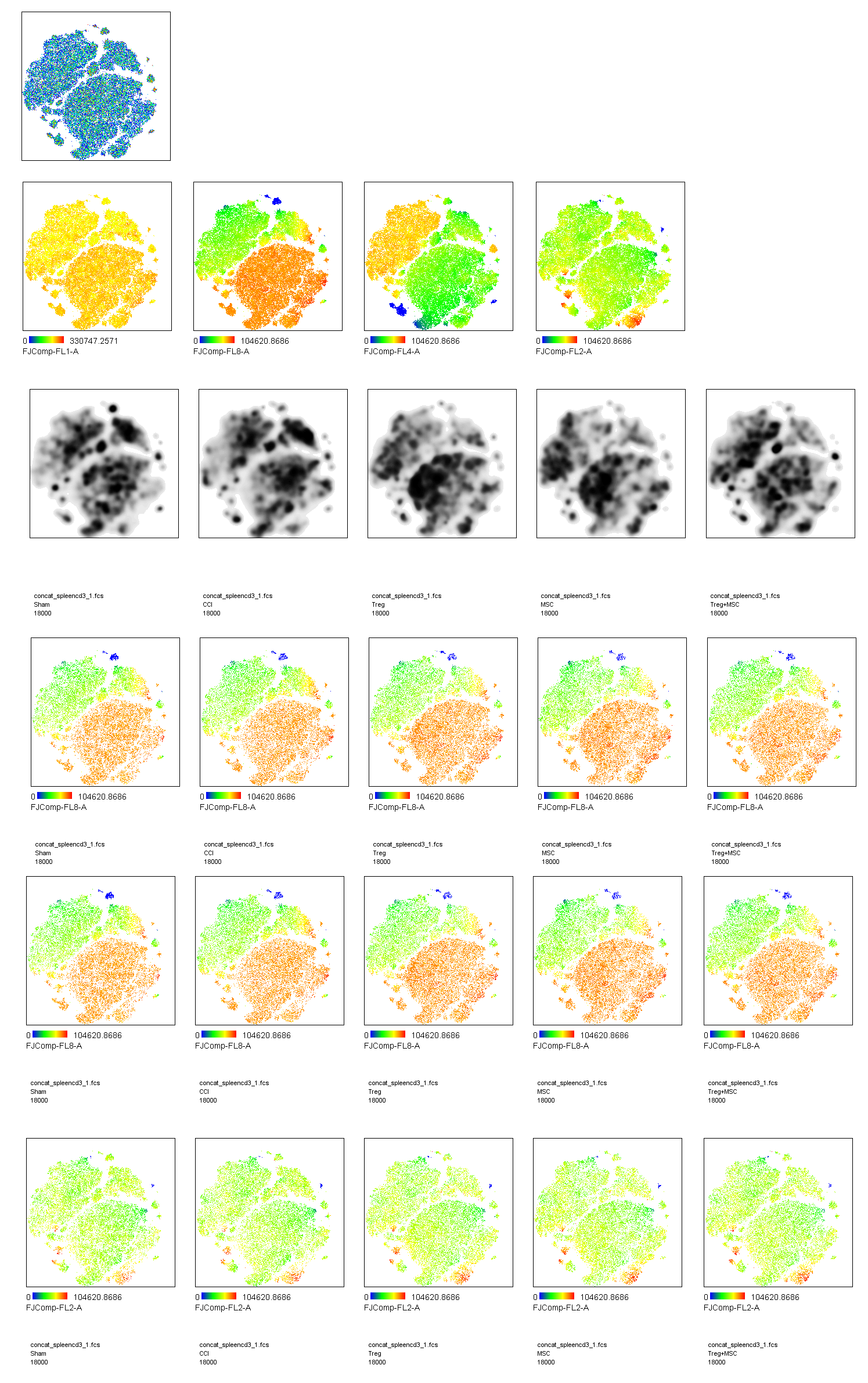

Supplement: S1 Data — (ZIP) [file pone.0251601.s004.zip › in vivo/11-26-20 96hr blood+spleen stats/treg+MSC 96hr spleen tsne CD3+ 11-26-19, 5-29-20.png]

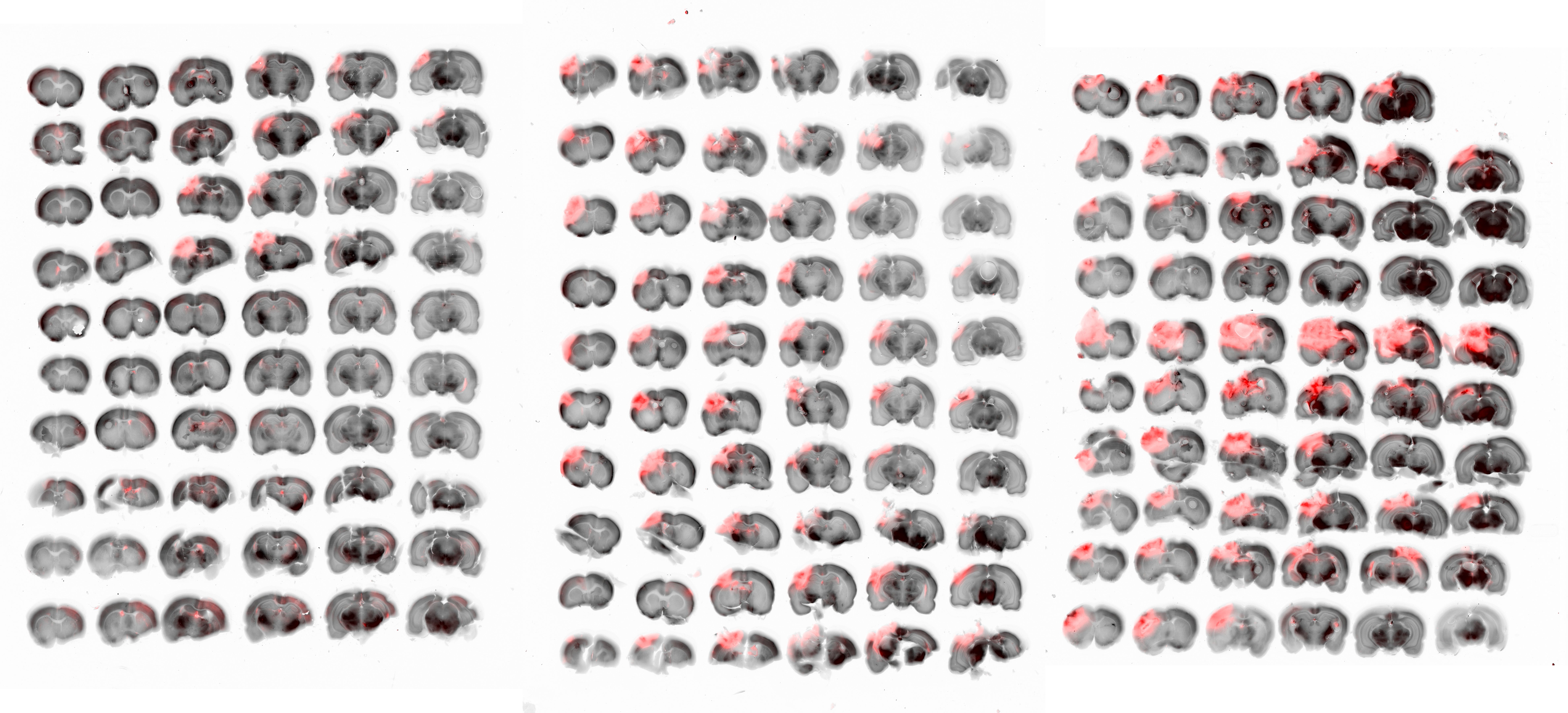

Supplement: S1 Data — (ZIP) [file pone.0251601.s004.zip › in vivo/BBB data 11-25-19/Composite on white.jpg]
