# Supplementary material for: Human-derived Treg and MSC combination therapy may augment immunosuppressive potency in vitro, but did not improve blood brain barrier integrity in an experimental rat traumatic brain injury model
Source: PLoS One. 2021 May 26;16(5):e0251601. doi: 10.1371/journal.pone.0251601 (PMC8153465; doi:10.1371/journal.pone.0251601)
Supplement: S1 Table — A table summary of the specific multicolor fluorescent antibody panel used to evaluate changes in rat immune cell populations for reference. (DOCX) [file pone.0251601.s003.docx]

| **Fluorochrome** | **Antibody** | **Clone** | **Supplier** |
| --- | --- | --- | --- |
| FITC | CD3 | 1F4 | Biolegend |
| PE | CD25 | OX-39 | Biolegend |
| PerCP | CD8a | OX-8 | Biolegend |
| PE Cy7 | CD11bc | OX-42 | BD |
| APC/AF 647 | RT1B | OX-6 | BD |
| APC Cy7 | CD4 | W3/25 | Biolegend |
| V450 | CD45RA | OX-33 | BD |

S1 Table: Multicolor Flow Cytometry Rat Immune Cell Panel
